# Diversity in Japanese Medical Research and Researchers

**DOI:** 10.31662/jmaj.2022-0213

**Published:** 2023-02-20

**Authors:** Soichiro Saeki, Misa Kusumoto

**Affiliations:** 1 Center Hospital of the National Center for Global Health and Medicine, Tokyo, Japan

**Keywords:** social determinants of health, health equity, populational health, aging society, racial disparities

2023 will be recognized as a special year for all involved in health equity, as we are to expect the “World Report on Social Determinants of Health Equity” to be submitted to the 76^th^ World Health Assembly, which is to be held beginning in May 2023 ^(1)^. This would be the first report to be released from the World Health Organization related to the social determinants of health (SDH) following the novel coronavirus disease (COVID-19) pandemic, as the last report was published in 2008 ^(1)^.

SDH is defined as “nonmedical factors that influence health outcomes” that influence “the unfair and avoidable differences in health status seen within and between countries” ^(1)^. Although many topics are covered in this agenda, such as education and healthcare access in Japan, this agenda has been generally focused on two primary topics: socioeconomic status and aging ^(2)^. This trend can be seen in the Japanese health promotion strategy “Health Japan 21,” which aims to increase healthy life expectancy of the Japanese. The population-based approach of conducting health screenings and interventions to prevent lifestyle diseases has propelled Japan to the forefront of the world’s aging societies.

However, the COVID-19 pandemic sparked new debates in SDH. Health disparities between races and ethnicities have been widely highlighted in various diseases, not limited to COVID-19 ^(3)^. While it is critical that such discussions do not exacerbate discrimination, they should encourage the Japanese community to promote diversity in research and researchers. Although Japanese populational and clinical studies typically report results consisting only of Japanese participants, as Japanese society becomes more diverse in race and ethnicity, they should also shed light on non-Japanese patients. Steps should be taken to eliminate the gender gap among researchers ^(4)^, as well as provide young researchers with the opportunity to publish their work ^(5)^. In addition to encouraging Japanese studies to be done from innovative viewpoints, new diversity among researchers and subjects would also help to perceive the medical needs of both healthcare staff and the general community from a number of perspectives.

Therefore, 2023 should be a watershed moment in Japan’s approach to SDH. Such action would pave the way for the Japanese medical society to provide adequate healthcare for all in a holistic and sustainable manner, while also encouraging the Japanese society as a whole to embrace diversity and accelerate the understanding of health among it. Everyone should be involved in shaping Japan’s future medical care system.

## Article Information

### Conflicts of Interest

None

### Acknowledgement

The authors are grateful to the colleagues for helpful discussions on this topic.

### Author Contributions

SS conceptualized the basic concept and MK critically revised the contents of the primary manuscript. Both authors edited the primary manuscript, read, and approved the final manuscript.

### Approval by Institutional Review Board (IRB)

Not applicable.
